# Differential expansion of T peripheral helper cells in early rheumatoid arthritis and osteoarthritis synovium

**DOI:** 10.1136/rmdopen-2022-002563

**Published:** 2022-10-21

**Authors:** William Murray-Brown, Yanxia Guo, Annabelle Small, Katie Lowe, Helen Weedon, Malcolm D Smith, Susan E Lester, Susanna M Proudman, Navin L Rao, Ling-Yang Hao, Sunil Nagpal, Mihir D Wechalekar

**Affiliations:** 1 Department of Rheumatology, Flinders University, Bedford Park, South Australia, Australia; 2 Discovery Immunology, Janssen Research and Development, Spring House, Pennsylvania, USA; 3 Rheumatology Research Group, Basil Hetzel Institute for Medical Research, Woodville South, South Australia, Australia; 4 Department of Rheumatology, Royal Adelaide Hospital, Adelaide, South Australia, Australia; 5 Discipline of Medicine, The University of Adelaide, Adelaide, South Australia, Australia; 6 Department of Rheumatology, Flinders Medical Centre, Bedford Park, South Australia, Australia

**Keywords:** Rheumatoid Arthritis, T Cells, T-Lymphocyte subsets

## Abstract

**Objectives:**

Programmed cell death protein 1 (PD-1)-expressing T cells are implicated in the pathogenesis of autoimmune inflammatory diseases such as rheumatoid arthritis. A subset of CXCR5^−^ T cells, termed T peripheral helper (Tph) cells, which drive B cell differentiation, have been identified in ectopic lymphoid structures in established rheumatoid arthritis synovial tissue. Here, we aimed to characterise these in treatment-naïve, early rheumatoid arthritis to determine whether these cells accumulate prior to fully established disease.

**Methods:**

Fresh dissociated tissue and peripheral blood mononuclear cell (PBMC) suspensions were stained with Zombie UV, followed by anti-CD45RO, PD-1, CD3, ICOS, CD8, CD4, CD20, CXCR5, TIGIT and CD38 antibodies prior to analysis. For histology, rheumatoid arthritis synovial sections were prepared for Opal multispectral immunofluorescence with anti-CD45RO, CD20, PD-1 and CXCR5 antibodies. Images were acquired on the Perkin Elmer Vectra V.3.0 imaging system and analysed using InForm Advanced Image Analysis software.

**Results:**

Flow cytometry revealed T cell infiltration in the rheumatoid arthritis synovium with differential expression of PD-1, CD45RO, ICOS, TIGIT and CD38. We observed a higher frequency of PD1^hi^CXCR5^−^ Tph in rheumatoid arthritis synovial tissue and PBMCs versus controls, and no significant difference in T follicular helper cell frequency. Microscopy identified a 10-fold increase of Tph cells in early rheumatoid arthritis synovial follicular and diffuse regions, and identified Tph adjacent to germinal centre B cells.

**Conclusions:**

These data demonstrate that PD-1^hi^ Tph cells are present in early rheumatoid arthritis, but not osteoarthritis synovium, and therefore may provide a target for treatment of patients with early rheumatoid arthritis.

**WHAT IS ALREADY KNOWN ON THIS TOPIC**

- While pathogenic PD-1^+^ T peripheral helper (Tph) cells have been implicated in the pathogenesis of established rheumatoid arthritis (RA), it remains unknown at what stage of RA Tph cells accumulate in the synovium, and whether they may present a target for early therapeutic intervention.

**WHAT THIS STUDY ADDS**

- We demonstrate differentially enriched Tph cells in the early, treatment-naïve RA synovium and periphery compared with healthy and osteoarthritis controls.
- Within the synovium, Tph cells far outnumbered T follicular helper cells, were located in close proximity to B cells in follicular and diffuse infiltrates, and their numbers correlated with B cell numbers.

**HOW THIS STUDY MIGHT AFFECT RESEARCH, PRACTICE OR POLICY**

- Our study, demonstrating the presence of pathogenic Tph cells in early RA synovium, lays the potential of using Tph-based therapeutic approaches for disease interception prior to fully established RA.

## Introduction

Programmed cell death protein 1 (PD-1) is an inhibitory immune checkpoint molecule with a prominent role in modulating T cell activity.^1^ Upon T cell activation, PD-1 is upregulated where it then induces inhibitory signals that limit the magnitude of the T cell response. This is elicited through interaction with the ligands PD-L1 and PD-L2.^2^ Normally, the expression of PD-1 is transient, and expression is downregulated during the resolution phase of the insult following clearing of antigen.^2^ However, in states of chronic antigen exposure, such as in cancer and autoimmunity, high PD-1 expression is sustained,^3^ and is typically associated with states of T cell dysfunction or exhaustion.^4^

PD-1-expressing T cells have been implicated in the autoimmune pathogenesis of chronic inflammatory diseases such as rheumatoid arthritis (RA)^5–8^ and Sjogren’s syndrome.^9^ Particularly, CD4^+^PD-1^+^CXCR5^+^ICOS^+^ T follicular helper (Tfh) cells secrete interleukin-21 and provide CD40:CD40L co-stimulatory signals to germinal centre (GC) B cells.^10^ This interaction allows for class switch recombination and somatic hypermutation of B cell receptor genes and the affinity maturation of the autoantibody responses to drive autoimmunity.^11^ In chronic autoimmune diseases, these cognate T–B cell interactions are thought to occur within ectopic lymphoid structures of inflamed tissues.^12^ In RA, this occurs within the synovial tissue (ST), where CD4^+^ICOS^+^PD-1^+^CXCR5^+^ Tfh cells were thought to provide important signals to autoreactive B cells in RA GCs within ectopic lymphoid structures.^13 14^ However, in 2017, Rao *et al*^7^ described a T cell subset devoid of CXCR5 expression, termed ‘T peripheral helper (Tph) cells’ present in the established RA ST.^7^ These cells exhibit an overlapping transcriptional profile to Tfh cells and can stimulate B cell differentiation to plasma cells, which secrete immunoglobulin.^7^

There has been described a ‘window of opportunity’ where superior clinical responses and increased potential for remission are observed when patients with RA are treated early.^15^ Critically, however, it remains unknown whether Tph cells accumulate in treatment-naïve early RA and contribute to disease establishment, and whether they may present a target for early therapeutic intervention. Here, we identify Tph cells and examine their localisation and interaction within the *early* RA ST and demonstrate differential enrichment of PD-1^hi^ Tph cells in ST from patients with treatment-naïve early RA when compared with patients with osteoarthritis (OA).

## Methods

### Human subjects

Arthroscopic synovial biopsies and venous blood were collected from four treatment-naïve patients with rheumatoid factor and anti-citrullinated protein antibody (ACPA)-positive early RA (<12 months of symptom onset and fulfilling the 2010 American College of Rheumatology/EULAR classification criteria). Blood was collected from three additional patients with early RA. Four non-inflammatory OA ST, five healthy control blood samples and three tonsil samples were collected as controls.

### Tissue preparation and flow cytometry

Fresh ST was dissociated using the human Tumor Dissociation Kit and the gentleMACS dissociator (Miltenyi Biotec) as per the manufacturer’s recommendations. Cells were filtered using 70 µm filters. Peripheral blood mononuclear cells (PBMCs) were purified using Lymphoprep as per the manufacturer’s recommendations (STEMCELL Technologies). PBMCs and ST cells were washed and counted prior to immunostaining. ST cell suspensions, along with matched PBMCs, were stained with Zombie UV (BioLegend) in serum-free phosphate buffered saline (PBS), followed by staining with anti-CD45RO-BUV395 (UCHL1), PD1-BV421 (EH12.1), CD3-PerCP/Cy5.5 (SK7), ICOS-PE (DX29), CD8-Alexa647 (RPA-T8), CD4-Alexa700 (SK3), CD20-APC/H7 (L27), TIGIT-PE/Cy7 (A15153G), CD38-BB515 (HIT2) (all from BD Biosciences) and CXCR5-PEDazzle (J252D4; BioLegend) in 2% fetal calf serum (FCS). Cells were resuspended in 2% FCS and acquired on a BD FACSAria Fusion flow cytometer. Data were analysed using FlowJo V.10.5.3 software (FlowJo, Ashland, Oregon, USA).

### Histology

The 4 µm thick formalin-fixed, paraffin-embedded (FFPE) RA ST and tonsil sections were prepared for Opal multispectral imaging as per the manufacturer’s recommendations (Perkin Elmer, Waltham, Massachusetts, USA). Briefly, FFPE sections were stained one by one with primary antibodies: anti-CD45RO (Dako; UCHL1), CD20 (Dako; L26), PD-1 (Abcam; NAT105) and CXCR5 (Atlas antibodies; HPA042432), each followed by secondary Horseradish peroxidase (HRP) amplification antibody staining and specific Opal reactive fluorophores. Primary antibody stripping prepared the section for the next primary antibody staining. Once all Opal targets were developed, 4′,6-diamidino-2-phenylindole (DAPI) nuclear staining was performed prior to image acquisition (Perkin Elmer Vectra V.3.0 automated imaging system). Images were processed and analysed using InForm Advanced Image Analysis software (Perkin Elmer, Waltham, Massachusetts, USA).

### Cell proximity analysis

Analysis of the cell segmentation files exported from InForm was performed using R V.4.0.0^16^ and the ‘phenoptr’ package.^17^ Cell co-localisation was examined using nearest neighbour distances. Briefly, the phenoptr ‘compute_all_nearest_distance’ command computes the distance to the nearest neighbour cell in each segment of the merged segmentation file for each of the included phenotypes (B cell, GC B cell, Tfh, Tph). The nearest neighbour distances from each Tph cell (n=57 994) or Tfh cell (n=2741), to either GC B cells or B cells were described further in Stata V.16 (StataCorp, Texas, USA), using kernel density plots of the distance distribution for each phenotype. Differences in distance distributions between cells were compared by the Kruskal-Wallis test.

### Statistical analysis

GraphPad Prism V.8.0 was used for statistical analysis. Mean differences were compared using t-tests or one-way analysis of variance followed by multiple comparison tests (for comparisons of three of more groups), and correlations were analysed using non-parametric, two-tailed Spearman correlations. P values of <0.05 were considered to be statistically significant.

## Results

### Tph cells are present within the ST and periphery of treatment-naïve early RA

**Figure 1:**
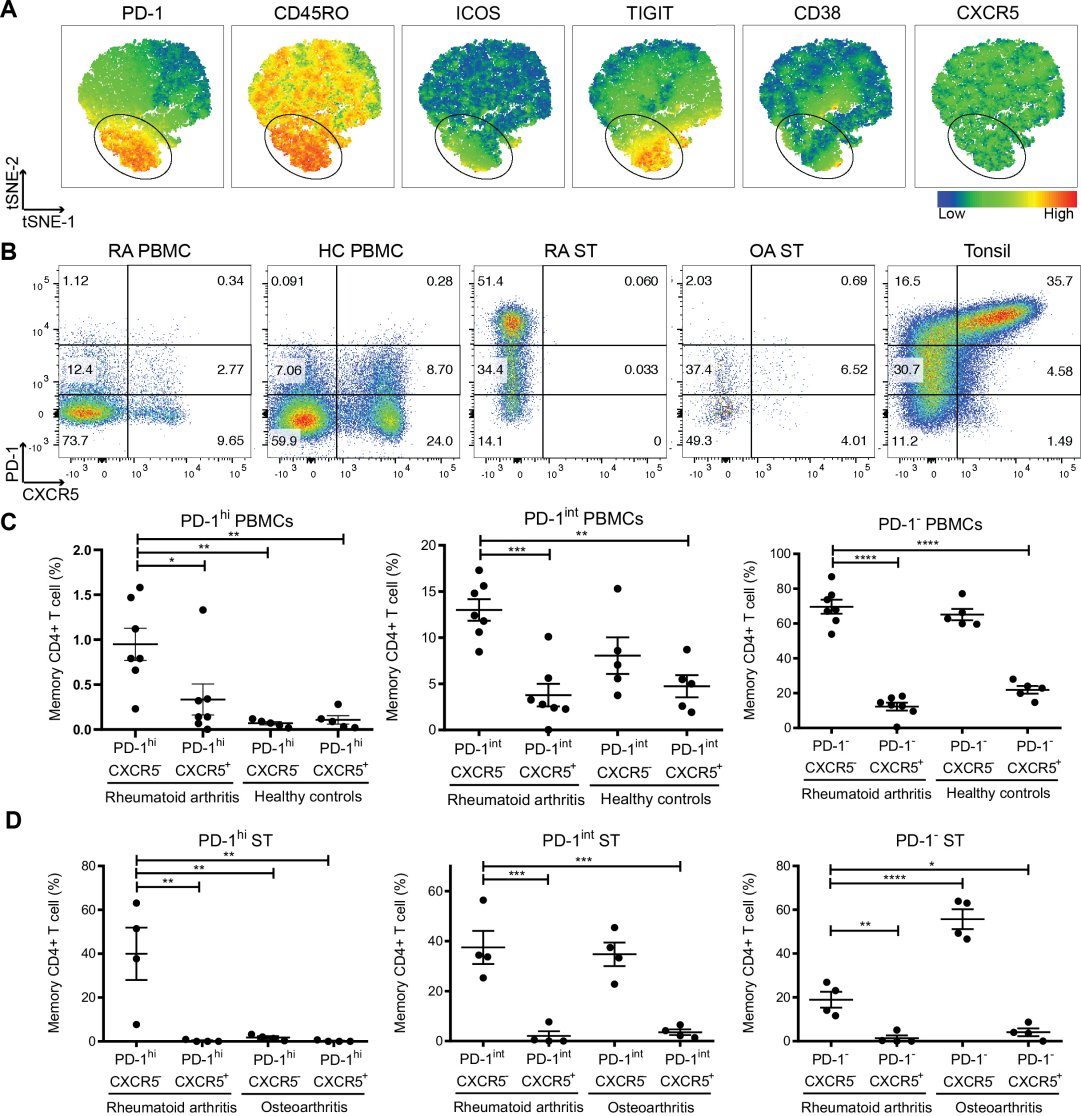
Expanded Tph cells in treatment-naïve early RA synovial tissue (ST) and peripheral blood. (A) viSNE plots of flow cytometry data from treatment-naïve early RA ST CD4^+^ T cells. Colour indicates cell expression levels of labelled marker, ring indicates PD-1^hi^ cells. (B and C) PD-1 and CXCR5 expressing cell frequency of peripheral blood memory T cells from RA (n=7) and healthy controls (HC, n=5). (B and D) PD-1 and CXCR5 expressing cell frequency of ST memory T cells from patients with RA (n=4) and OA (n=4). Data are presented as mean±SD (C, D). *P<0.05, **p<0.01, ***p<0.001, by one-way analysis of variance test (ANOVA) followed by Tukey’s multiple comparison tests. OA, osteoarthritis; PBMC, peripheral blood mononuclear cell; PD-1, programmed cell death protein 1; RA, rheumatoid arthritis; Tph, T peripheral helper; viSNE, visualisation using t-Distributed Stochastic Neighbor Embedding.

We analysed CD4^+^ T cell populations present in seropositive, treatment-naïve early RA PBMC samples (n=7, table 1) using flow cytometry. CD4^+^ T cells were gated (online supplemental figure 1), and visualisation using t-Distributed Stochastic Neighbor Embedding (viSNE)^18^ demonstrated heterogeneous T cell infiltration into the ST with differential expression of PD-1, CD45RO, ICOS, TIGIT and CD38 (figure 1A).

10.1136/rmdopen-2022-002563.supp1 Supplementary data

**Table 1:** Clinical characteristics of patients with seropositive, treatment-naïve, early RA included in the study

	Synovial tissue and blood donors (n=4)				Blood donors only (n=3)		
Patient	1	2	3	4	5	6	7
Age	54	37	73	45	66	60	46
Sex	M	M	M	F	F	F	F
Disease duration (weeks)	11	16	11.7	12.6	48	12	16.6
CRP (mg/L)	14	7.9	33	55.8	1.42	8.3	49.4
Disease Activity Score	8.06	5.26	6.15	6.55	3.96	4.8	5.4
CCP (mg/L)	40	92	196	>300	>300	>300	>300
Erythrocyte sedimentation rate	115	27	79	59	10	18	104

CCP, cyclic citrullinated peptide antibody; CRP, C reactive protein; RA, rheumatoid arthritis.

We observed a higher frequency of PD-1^hi^CXCR5^−^ Tph cells in the peripheral blood of patients with early RA compared with healthy controls (HC) (figure 1B, C), consistent with previous observations in patients with established RA.^7^ PD-1^hi^CXCR5^+^ Tfh cells were comparable between RA and HC, as were PD-1^int^ and PD-1^−^ CD4^+^ T cells, regardless of CXCR5 expression (figure 1C). We next assessed for correlation of Tph and Tfh cell numbers with clinical parameters; however, due to low sample size, we did not observe any correlation between Tph numbers and Disease Activity Score (DAS28) (p=0.8587), C reactive protein (CRP) (p=0.7571), cyclic citrullinated peptide antibody (CCP) (p=0.9282) or erythrocyte sedimentation rate (ESR) (p=0.9857). Likewise, Tfh numbers did not correlate with DAS28 (p=0.9635), CRP (p=0.3536), CCP (p=0.2135) or ESR (p=0.7131).

**Figure 2:**
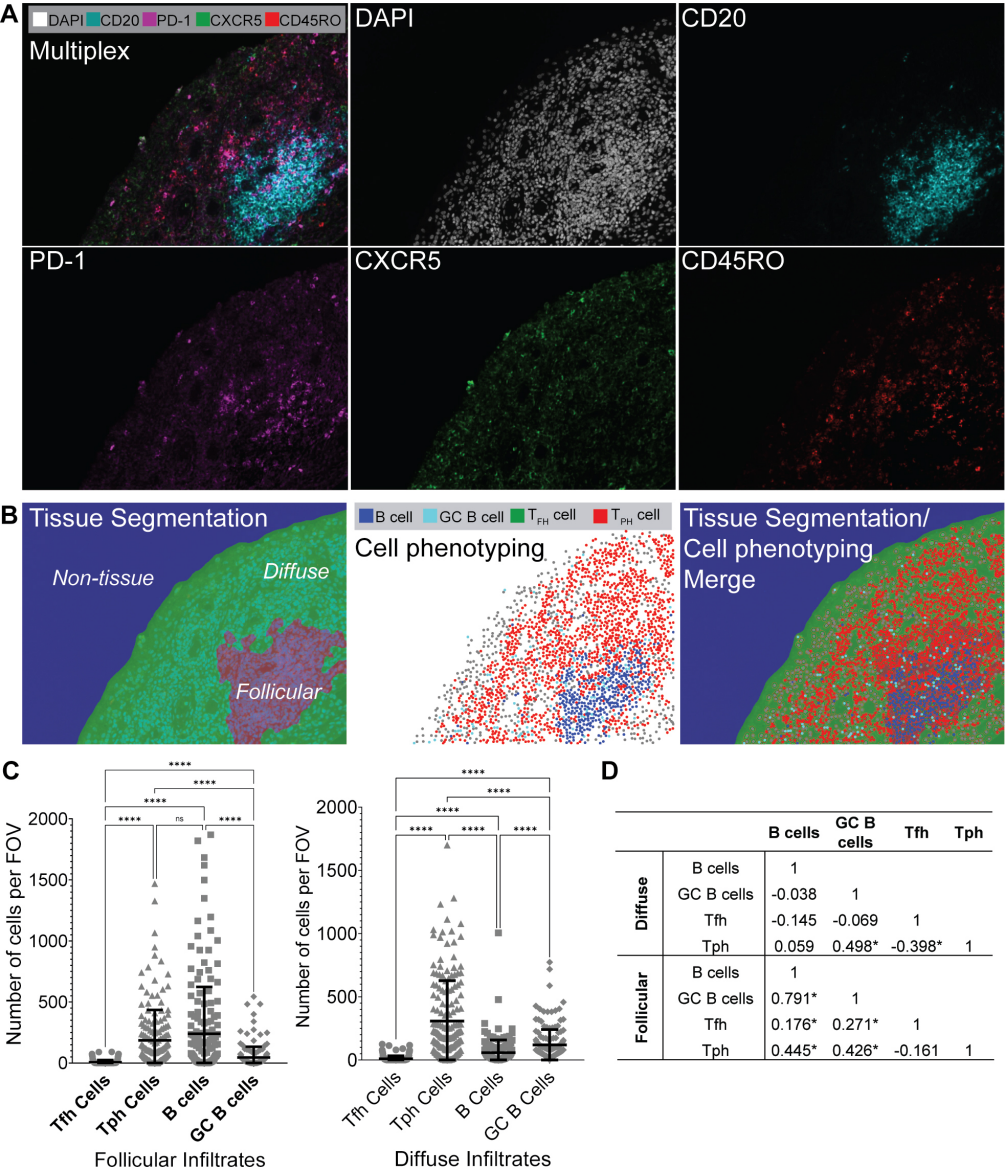
Tph cells are enriched in lymphoid aggregates and in proximity to germinal centre (GC) B cells. (A) Immunofluorescence microscopy of RA synovium at ×20 magnification, showing DAPI, CD20, PD-1, CXCR5 and CD45RO staining. Multiplexed image is shown (upper left), and each individual stain is shown. (B) Tissue segmentation (left), phenotyping (middle) and merged image (right) as determined by InForm imaging software, ×20 magnification. (C) Numbers of Tfh, Tph, B cells and GC B cells per field of view (FOV) within follicular infiltrates (left) and diffuse infiltrates (second from left). Data are presented as mean±SD, and each dot point represents an individual identified cell. ns=not significant; ****p<0.0001, by one-way ANOVA followed by Tukey’s multiple comparison tests. Data are representative of n=4 experiments. (D) Spearman rank correlations between Tfh, Tph, B cell and GC B cell numbers, *p<0.05. ANOVA, analysis of variance; PD-1, programmed cell death protein 1; RA, rheumatoid arthritis; Tfh, T follicular helper; Tph, T peripheral helper.

We next analysed the CD4^+^ T cell populations in disaggregated early RA ST biopsy samples (n=4) (figure 1B and D). Although we observed a distinctive CXCR5^−^ T cell population in the ST of all four patients with early RA, there were few detectable CXCR5^+^ Tfh cells in the same tissue (figures 1B and 2D and online supplemental figure 1). Tfh and Tph cells were present in the ST of patients with OA, as in healthy tonsil (figure 1B and online supplemental figure 2). Interestingly, there were significantly higher numbers of PD-1^hi^CXCR5^−^ Tph cells in early RA ST compared with their OA counterparts (figure 1D). These data suggest that PD-1^hi^ Tph cells accumulate in the ST of early RA prior to fully established disease.

### Tph cells populate ST ectopic lymphoid structures

Immunofluorescence microscopy using the Opal multiplexed workflow identified CD45RO-expressing memory T cells with bright PD-1 expression in all four seropositive early RA intact ST samples (representative images shown in figure 2A). InForm Image Analysis software allowed us to identify tissue segmentation as diffuse versus follicular regions of the ST and identify specific cell types within the tissue (figure 2B). We examined four cell populations of interest: B cells, (CD20^+^CD45RO^−^CXCR5^−^); GC B cells, (CD20^+^CD45RO^−^CXCR5^+^); Tfh cells (CD20^−^CD45RO^+^CXCR5^+^PD-1^+^) and Tph cells (CD20^−^CD45RO^+^CXCR5^−^PD-1^+^). Using this analysis, we quantified Tfh, Tph, B cells and GC B cells per field of view (FOV) in early, treatment-naïve RA ST sections (n=4) (figure 2C). We observed significantly higher numbers of Tph cells per FOV compared with Tfh cells in follicular (p<0.0001) and diffuse (p<0.0001) infiltrates. We additionally observed higher numbers of Tph cells than GC B cells in follicular and diffuse infiltrates, higher Tph cells than B cells in diffuse infiltrates, and no significant difference between Tph and B cells in follicular infiltrates (p=0.3125). Spearman rank correlations (figure 2D) identified significant positive correlation between numbers of B cells and GC B cells, Tfh, and Tph cells, between GC B cells and Tfh and Tph cells, and negative correlation between Tfh and Tph cells in follicular infiltrates. In diffuse infiltrates, we observed significant positive correlation between Tph and GC B cells, and negative correlation between Tph and Tfh cells.

### Tph and Tfh cells co-localise with B cells in treatment-naïve early RA ST

**Figure 3:**
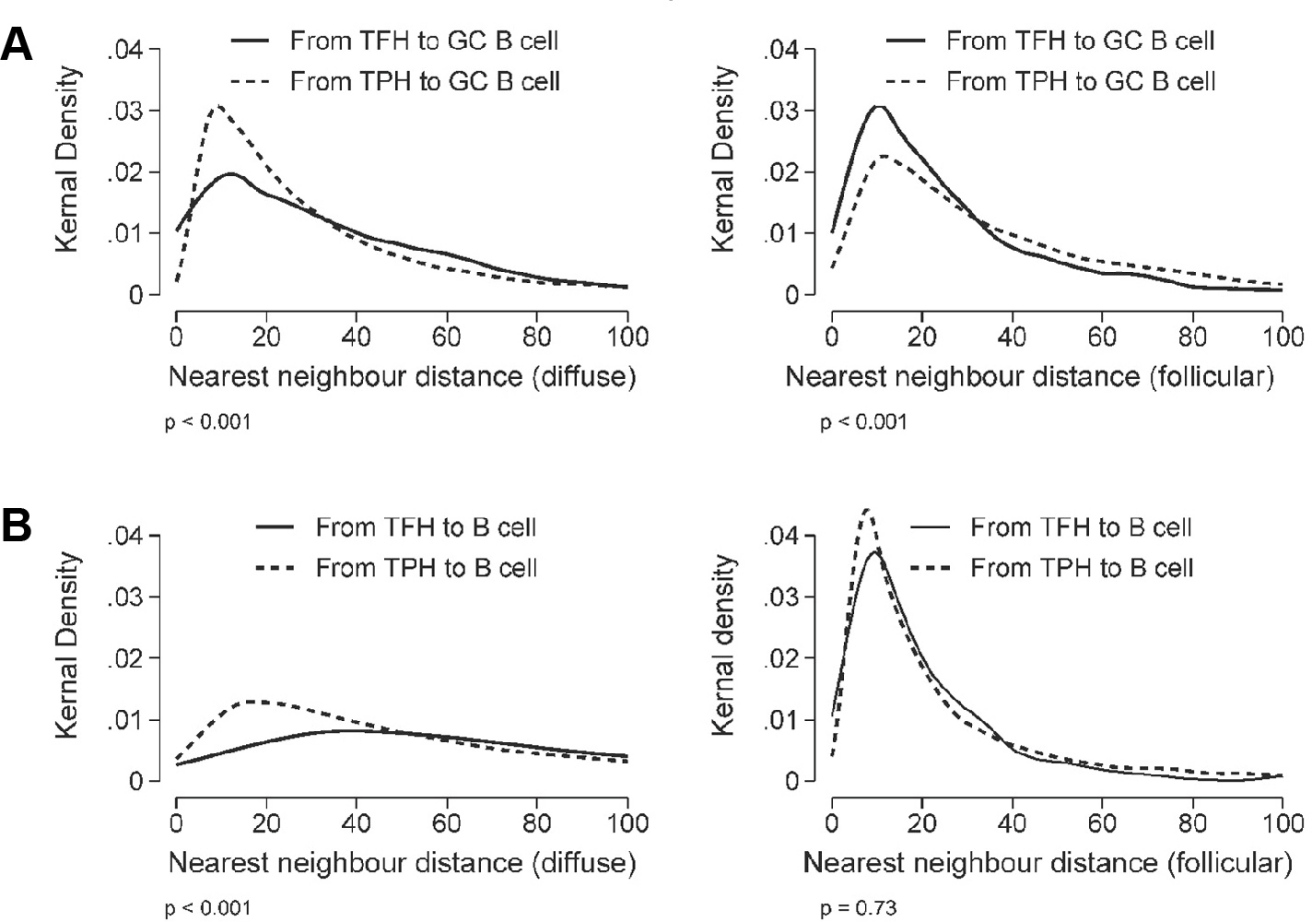
Tph cells are located in close proximity with B cells and GC B cells in diffuse and follicular infiltrates. Kernal density distributions of nearest neighbour distances (in microns) from Tph (solid lines) or Tfh (dashed lines) cells to either GC B cells (A) or B cells (B) in treatment-naïve, early RA synovium with diffuse and follicular infiltrates. The distance distributions were compared between Tfh and Tph cells using the Kruskal-Wallis test. GC, germinal centre; RA, rheumatoid arthritis; Tfh, T follicular helper; Tph, T peripheral helper.

Following InForm cell phenotype analysis, we measured the distance between identified Tph and Tfh cells and B cells in diffuse and follicular regions of early RA ST (n=4) (figure 3). Nearest neighbour analysis revealed that GC B cells (figure 3A) and B cells (figure 3B) were present within 20 μm of Tph and Tfh cells in both diffuse and follicular regions. We observed a higher probability that GC B cells were located in proximity with Tph cells in diffuse regions (figure 3A, left); while in follicular regions, there was a higher probability that GC B cells were in proximity with Tfh cells (figure 3A, right).

## Discussion

Here, we demonstrate Tph cell, rather than Tfh cell, accumulation in the ST of patients with treatment-naïve, early RA. These Tph cells can be divided into three subsets based on surface PD-1 expression. PD-1^hi^ Tph cells were differentially expanded in RA versus OA synovium, whereas PD-1^int^ Tph cells were expressed both in RA and OA ST (figure 1D). Further, Opal multispectral imaging (figures 2 and 3) revealed synovial Tph cells located in proximity to B cells and GC B cells, suggesting cellular interactions. While we also detected CXCR5^+^ Tfh cells in proximity with B cells and GC B cells (figure 3), the numbers of these detected within the ST were significantly lower than Tph cells (figures 1D and 2C). While a potential limitation of enzymatic tissue digestion is the cleavage of cell surface receptors,^19^ this is unlikely to be causing our observation of low Tfh cell numbers. As demonstrated in our control tonsil digestions (figure 1B), CXCR5^+^ Tfh cells were abundant and within intact tissue sections, Tfh cell numbers were similarly observed to be low in both follicular and diffuse regions (figure 2C). Thus, the low levels of Tfh cells observed within the RA ST in disaggregated samples likely reflect the cellular composition of the ST, rather than enzymatic cleavage of the surface receptor CXCR5 (figure 1B). Finally, the location of Tph cells was not restricted to diffuse or follicular regions of the tissue, indicating their ability to provide B cell help both within lymphoid aggregates and in the diffuse inflamed tissue. Meanwhile, we observed limited numbers of Tfh cells even in follicular regions, likely reflecting their specialised niche within lymphoid organs.^20 21^ Thus, together, these data suggest that Tph rather than Tfh cells provide B cell help within the early RA ST, potentially driving disease progression.

Tfh numbers within the lymphoid organs of patients with early RA have been demonstrated to correlate with B cell numbers within the same site.^21^ In our study, we assessed Tph and Tfh cell numbers in the early RA ST and identified positive correlation between Tph and Tfh cells with B cells and GC B cells in follicular regions, and a negative correlation between Tph and Tfh cells in diffuse regions (figure 2D). This, together with our observation of abundant Tph cells (figure 2C), suggests that adaptive immune cell interactions involving Tph cells may be increased locally within the early RA ST. In the GC of lymphoid organs, help provided by Tfh cells is usually limited as they are outnumbered by B cells, therefore promoting affinity maturation of B cells with the highest affinity for antigen.^22^ Our finding of abundant Tph cells further suggests that in the inflamed early RA ST, B cell help is readily available, potentially allowing affinity maturation of autoreactive B cell clones with low affinity, driving production of autoantibodies locally in the inflamed tissue.

The current standard treatment regimen for patients with RA is inadequate in preventing progression from early to established disease. Our previous study has shown the existence of CD4^+^PD-1^+^ T cells in synovial biopsies of patients with early and established RA.^8^ The present study expands on this, further demonstrating the dominant presence of CD4^+^PD-1^+^CXCR5^−^ Tph cells over Tfh cells in early RA ST. Furthermore, our study elaborates the cognate interaction between Tph cells and GC B cells in the synovial tissue, suggesting the key role of this interaction to produce autoantibodies in driving disease progression and tissue damage. Thus, our findings of PD-1^hi^ Tph cell accumulation within the early RA ST suggest that PD-1^hi^ Tph cells may be targeted in early disease to prevent further progression.

Immune checkpoint blockade, particularly in the form of therapeutics that interfere with PD-1:PD-L1 signalling, has revolutionised cancer treatment.^23–25^ However, by inhibiting the natural mechanisms limiting the magnitude and duration of the T cell response, these inhibitors are associated with a range of immune-related adverse events (irAEs),^26^ including inflammatory arthritis resembling RA.^2 27^ Our findings of Tph expansion in early, treatment-naïve RA suggest a role of these cells in driving the initial phase of disease. Thus, inflammatory arthritis as an irAE in patients with cancer treated with PD-1-targeting therapeutics may be a manifestation of enhanced Tph cell function.

However, despite the development of irAE following PD-1 pathway blockade,^28^ PD-1 may yet serve as a therapeutic target in the early stages of autoimmunity through pathway agonism.^6^ Our demonstration here of PD-1^hi^ Tph cells in early RA indicates that compared with existing therapeutics, targeting T cells agonistically through PD-1 may inhibit both T cell-mediated and autoreactive B cell-mediated tissue damage during early RA disease progression. Tph cells from patients with established RA have previously been demonstrated to promote plasma cell differentiation and induce IgG production.^7^ Thus, depletion and/or agonism of PD-1^hi^ Tph cells in situ may limit local autoantibody production. Therefore, therapeutic targeting of the PD-1 pathway locally in the RA ST has the potential to affect both B and T cells in the treatment of early RA and thus, may provide deeper responses and higher remission rates than the current generation of therapeutics. Indeed, investigation into the utility of bispecific antibodies to treat RA is already underway, and early studies have demonstrated successful targeting to the ST.^29^

In summary, our results indicate that PD-1 is a potential target for treatment of early RA and may prevent the progression of the disease to fully established RA.
