# Supplementary material for: Differential expansion of T peripheral helper cells in early rheumatoid arthritis and osteoarthritis synovium
Source: RMD Open. 2022 Oct 21;8(2):e002563. doi: 10.1136/rmdopen-2022-002563 (PMC9594577; doi:10.1136/rmdopen-2022-002563)
Supplement: Supplementary data [file rmdopen-2022-002563supp001.pdf]

Supplementary information

Differential Expansion of T Peripheral Helper Cells in early Rheumatoid Arthritis and Osteoarthritis Synovium

William Murray-Brown[1], Yanxia Guo[2], Annabelle Small[1], Katie Lowe[1], Helen Weedon[1], Malcolm D. Smith[1], Susan Lester[3], Susanna Proudman[4,5], Navin L. Rao[2], Ling-Yang Hao[2], Sunil Nagpal[2], Mihir D. Wechalekar[1,6*]

[1]Department of Rheumatology, Flinders University, Bedford Park, South Australia, 5042, Australia
[2]Discovery Immunology, Janssen Research, 1400 McKean Road, Spring House, PA, 19477, USA
[3]Rheumatology Research Group, Basil Hetzel Institute, Adelaide, South Australia, 5011, Australia
[4]Department of Rheumatology, Royal Adelaide Hospital, Adelaide, Australia
[5]Discipline of Medicine, University of Adelaide, Adelaide, Australia.
[6]Department of Rheumatology, Flinders Medical Centre, Bedford Park, South Australia, 5042, Australia

**Correspondence:*
Associate Professor Mihir Wechalekar
mihir.wechalekar@sa.gov.au

Contents of Supplementary Information:

1. Supplementary Figure 1
2. Supplementary Figure 2

Murray-Brown W, *et al. RMD Open* 2022; 8:e002563. doi: 10.1136/rmdopen-2022-002563

Figure legends

Supplementary Figure 1. Detection of PD-1 and CXCR5 expressing cells in disaggregated ST biopsy samples by flow cytometry. Gating strategy to identify PD-1 and CXCR5 expressing memory $CD4^+$ T cells by flow cytometry and gating of $CD4^+$ T cells prior to t-SNE analysis. Cells were gated for lymphocytes, singlets, live cells, $CD3^+$ $CD20^-$ cells and $CD4^+$ $CD8^-$ cells prior to t-SNE or further gating for $CD45RO^+$ memory T cells and detection of PD-1 and CXCR5 expressing populations via 2-D dot plots.

Supplementary Figure 2. Differential gene expression of CD4+ T cells identified by viSNE analysis. (A) viSNE plots of flow cytometry data from tonsil control and RA synovial tissue $CD4^+$ T cells. (B) viSNE plots of flow cytometry data from RA and, (C) healthy control peripheral blood $CD4^+$ T cells. Colour indicates cell expression levels of labelled marker. Ring indicates PD-1^{hi} cells.

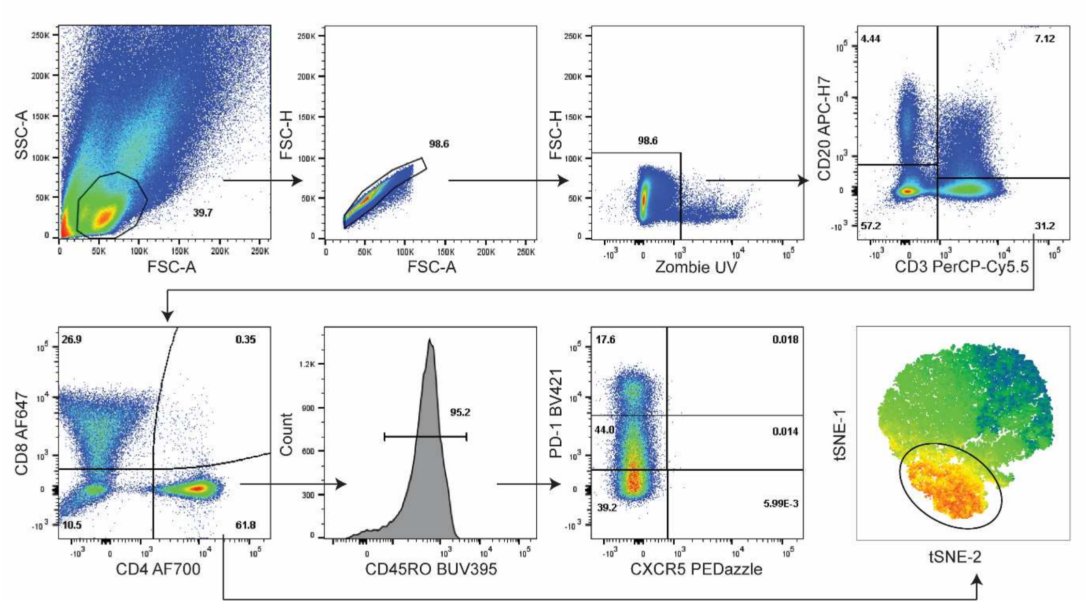

Supplementary Figure 1.

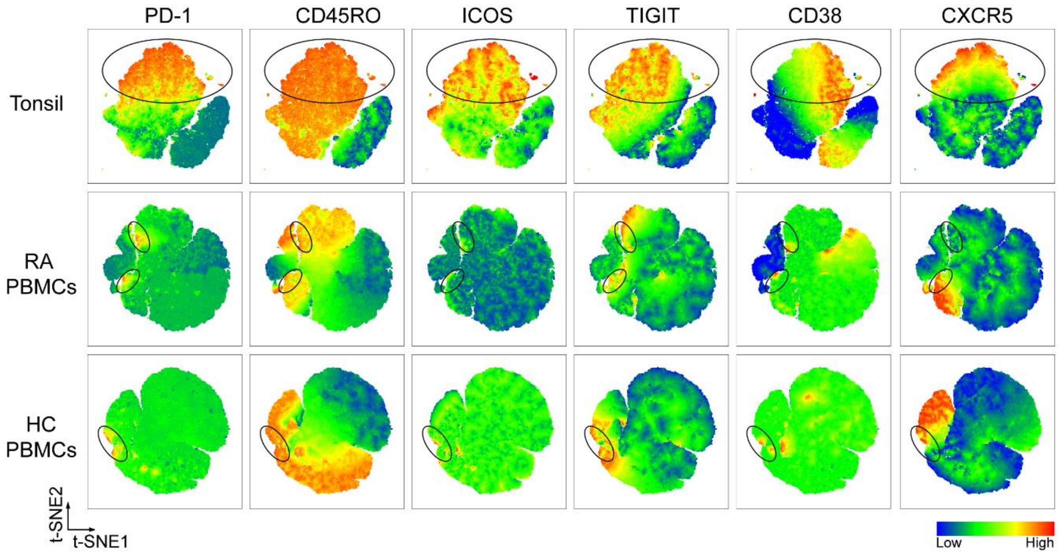

Supplementary figure 2.
